# Rapid evolutionary response to a transmissible cancer in Tasmanian devils

**DOI:** 10.1038/ncomms12684

**Published:** 2016-08-30

**Authors:** Brendan Epstein, Menna Jones, Rodrigo Hamede, Sarah Hendricks, Hamish McCallum, Elizabeth P. Murchison, Barbara Schönfeld, Cody Wiench, Paul Hohenlohe, Andrew Storfer

**Affiliations:** 1 School of Biological Sciences, Washington State University, Pullman, Washington 99164-4236, USA; 2 School of Zoology, University of Tasmania, Private Bag 5, Hobart, Tasmania 7001, Australia; 3 Department of Biological Sciences, Institute for Bioinformatics and Evolutionary Studies, University of Idaho, 875 Perimeter Drive, Moscow, Idaho 83844-3051, USA; 4 School of Environment, Griffith University, Nathan Campus, 170 Kessels Road, Nathan, Queensland 4111, Australia; 5 Department of Veterinary Medicine, University of Cambridge, Madingley Road, Cambridge CB3 0ES, UK

## Abstract

Although cancer rarely acts as an infectious disease, a recently emerged transmissible cancer in Tasmanian devils (*Sarcophilus harrisii*) is virtually 100% fatal. Devil facial tumour disease (DFTD) has swept across nearly the entire species' range, resulting in localized declines exceeding 90% and an overall species decline of more than 80% in less than 20 years. Despite epidemiological models that predict extinction, populations in long-diseased sites persist. Here we report rare genomic evidence of a rapid, parallel evolutionary response to strong selection imposed by a wildlife disease. We identify two genomic regions that contain genes related to immune function or cancer risk in humans that exhibit concordant signatures of selection across three populations. DFTD spreads between hosts by suppressing and evading the immune system, and our results suggest that hosts are evolving immune-modulated resistance that could aid in species persistence in the face of this devastating disease.

## Abstract (web summary)

A recently emerged infectious cancer has caused the near extinction of the Tasmanian devil, but some populations persist. Here, Epstein *et al*. provide evidence for possible resistance via rapid evolution in two genomic regions that contain cancer-related immune response genes.

## Graphical abstract

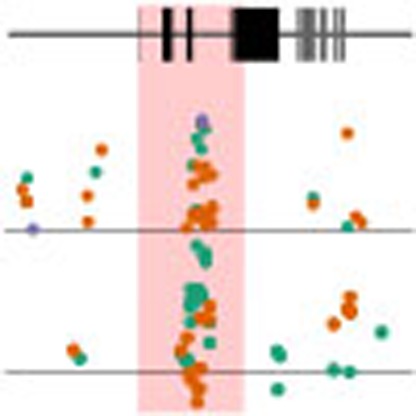

Emerging infectious diseases are increasingly implicated in population declines and even species' extinctions^1^,^2^. One of the most fascinating recent emerging infectious disease discoveries has been that of Tasmanian devil facial tumour disease (DFTD), a transmissible cancer^3^,^4^. Remarkably, DFTD is caused by a directly transmissible cell line^3^, and only two other such cancers are known outside the laboratory. The canine transmissible venereal tumour (CTVT) is at least 11,000 years old and is generally not fatal to domesticated dogs^4^. A recently discovered cancer affecting marine bivalves along the east coast of North America is apparently causing declines, but the extent of losses is not known^5^. In contrast, DFTD has caused population declines as great as 95% and an overall decline of over 80% of Tasmanian devils (*Sarcophilus harrisii*) in only 20 years^1^,^6^. First detected in northeastern Tasmania in 1996, DFTD has been nearly 100% fatal and will soon encompass the entire geographic range of *S. harrisii*, leaving no uninfected wild populations. Very recently, a second transmissible cancerous cell line (DFT2) has been described in Tasmanian devils^7^. This tumour results in similar symptoms, but has an independent origin from a male devil (the original DFT came from a female) and a different karyotype^7^. Given the recent discovery of DFT2 (2014), its population level and evolutionary impacts on Tasmanian devil populations are currently unknown. Nonetheless, the appearance of DFT2 raises the hypothesis that a combination of the unique life history and historical demography of the Tasmanian devil has created a ‘perfect storm' for the evolution of transmissible cancers.

As the largest remaining marsupial carnivore, Tasmanian devils are nocturnal, highly social, and extremely aggressive toward one another. The frequency-dependent transmission of DFTD, which is spread by biting during social interactions, has led to predictions of Tasmanian devil extinction on the basis of epidemiological models^1^,^8^. Further, Tasmanian devils have experienced historical population bottlenecks, and evidence suggests overall low genetic variability across the entirety of their current geographic range^9^. Indeed, the lack of genome-wide variation in Tasmanian devils, combined with irregular tumour MHC expression and downregulation of host MHC by DFTD, have led to what appears to be universal susceptibility^10^. However, populations predicted to be extinct by now continue to persist in low numbers.

The extremely strong selection imposed by DFTD could result in devil extinction, tumour extinction, or some type of stable or unstable equilibrium between the host and pathogen. Here, we present results utilizing a unique opportunity to study devil genomic responses to DFTD across both time and space. We have replicated sampling across multiple populations before DFTD arrived and at several time points following initial epizootics. To test for patterns of selection across the Tasmanian devil genome, we generated >90,000 single-nucleotide polymorphisms (SNPs) by sampling >800,000 loci using RAD-seq (Restriction-site Associated DNA sequencing^11^) from 294 individuals collected from three locations across Tasmania (Freycinet, Narawntapu and West Pencil Pine; Fig. 1). We identify two genomic regions with evidence of strong selection, both containing genes related to immune function or cancer risk, suggesting that Tasmanian devils are evolving resistance to DFTD.

## Results

### Identification of candidate regions

We identified two chromosomal regions (in chromosomes 2 and 3; Fig. 2a,g) that appear to be under strong selection based on the following analyses. First, these regions contained SNPs with allele frequency changes in the top 2.5% when comparing pre- and post-disease samples (Fig. 2b,g; see Supplementary Fig. 1 for examples of allele frequency change). Then, a 200 kb window was drawn around each of those SNPs (100 kb on either side) based on strong linkage disequilibrium (LD) at this scale (Supplementary Fig. 2). The two candidate regions are where windows from all three populations overlapped (see Fig. 2, gray bars). SNPs occurred approximately every 33 kb across the 3 Gb Tasmanian devil genome, providing an average of at least one SNP per linkage group. Weak or non-existent correlations in allele frequency change between populations throughout the remainder of the genome suggest that the three populations are otherwise evolving independently (Supplementary Table 1). Within the two candidate regions, we further tested for selection using four different sets of analyses.

First, we tested for changes in the extent of LD in the two candidate regions in pre-DFTD and post-DFTD samples using the Rsb statistic^12^. LD should increase in genomic regions near a variant that has recently experienced strong positive selection^12^. All genes in the chromosome 3 candidate region were within 100 kb of SNPs in the top 2.5% of Rsb values in two of three populations (Freycinet and Nawrantapu). There are also SNPs with strongly positive Rsb values within and near the candidate region on chromosome 2 (Fig. 2c,l). Across the genome as a whole, however, there is little correlation between the Rsb statistic and allele frequency change (Supplementary Table 1). To test for concordance among changes in allele frequencies and LD for signatures of selection in our candidate regions, we adapted a composite statistic^13^ using Fisher's method for combining *P* values applied to 100 kb sliding windows (see the ‘Methods' section). The windows that overlapped the candidate regions were in the top 3% of all windows tested across the entire genome, and had combined *P* values of 0.015 and 0.004 for the chromosome 2 and 3 regions, respectively.

Second, we investigated the directionality of allele frequency change in the three populations. Within the chromosome 2 candidate region, allele frequency changes are in the same direction in all three populations at many SNPs (Fig. 2e). This pattern is not seen as clearly in the chromosome 3 region (Fig. 2j), which may indicate that the pre-DFTD LD relationships between the markers we sequenced and the selected alleles are different in different populations, or that the causative variants are not the same in all the populations.

Third, five of the seven genes in these regions contain protein-coding genes associated with cancer risk or immune system function in other mammals (Table 1). We tested the likelihood that this result could have occurred by chance because there are a large number of genes across the mammalian genome associated with these functions. We sampled 1,000 pairs of 200 kb regions around randomly selected SNPs in our data set, and only 25% of such regions contained one or more genes with Gene Ontology^14^ terms ‘immune response,' ‘cell proliferation,' or ‘cell death' (our candidate regions contained both an immune response and a cell death GO annotation). Consequently, our result (two out of seven based on GO terms or five out of seven based on manual annotation) is unlikely to have been found by chance. The five genes include the following. First, CD146, or melanoma cell adhesion molecule, is a cell surface protein that has a wide range of functions in signalling and cell adhesion that include regulation of the immune system and inflammatory response^15^. Second, Thy-1 cell surface antigen (*THY1* or *CD90*) is a membrane protein involved in the regulation of a variety of cell–cell interactions including immune system function, cell adhesion and tumour suppression^16^. Third, mutations in Cbl-proto-oncogenes (homologous to *Cbl-c*, or *CBL2*) are associated with changes in cancer risk in humans and mutations in related genes increase the ability to fight off tumours in mice^17^. Fourth, ubiquitin-specific peptidase 2 (*USP2*) is a target of cancer treatments because it is involved in cell-cycle regulation and particularly *p53*, which regulates apoptosis^18^. Fifth, cereblon (*MRT2*) is a target for drugs used to treat myeloma^19^.

Fourth, we used a time series analysis to test for relative fitness advantages of SNPs in the two genomic regions by fitting a model of allele frequency change^20^ to the data (Fig. 2d,i). The point estimates for the mean selective advantage of increasing alleles in the chromosome 2 candidate region are 29% in Freycinet (point estimate range: 18–43%), 28% in Narawntapu (0.5–101%) and 19% in West Pencil Pine (1–51%); and for chromosome 3, the estimates were 16% in Freycinet (0–31%), 38% in Narawntapu (0–121%) and 30% in West Pencil Pine (2–107%).

## Discussion

We present strong evidence for an evolutionary response to selection imposed by DFTD in two small candidate genomic regions based on multiple lines of evidence. Allele frequency changes in SNPs that were in the top 2.5% pre- and post-DFTD overlapped in these two regions in all three populations. Given our method of detecting candidate gene regions and the extent of LD (Supplementary Fig. 2), this represents sufficient coverage to detect loci under selection across approximately one-sixth of the genome. Within these regions, there are strong increases in LD indicative of selective sweeps expected to occur in genomic regions near a variant that has recently experienced strong positive selection^12^. Further, our composite statistic shows that the consistency of these two analyses was statistically unlikely to have occurred by chance. Time series analyses bolster these results by suggesting allele frequency changes confer a fitness advantage to devils with the selectively favoured allele.

Importantly, five of seven genes in the two candidate regions are related to cancer or immune function in other mammals. One gene, cereblon, a myeloma treatment target, is found in strong linkage with the chromosome 2 region. Although there are four candidate genes in chromosome 3, it is likely that only one gene is the cause of the signatures of selection due to the strength of LD at this scale. The two most probable candidates are *CD146* and *THY1* because they act as immune system regulators and are involved in cell–cell communication and cell adhesion. Given that DFTD is likely infectious due to immune system evasion via improper expression of its own MHC, as well as downregulation of MHC expression in the devil host^10^, the functions of these genes suggest that the devil immune system may be adapting to be able to recognize tumour cells. Five of the candidate genes are consistent with those that manipulate host immunity by CTVT^21^. Thus, although the Tasmanian devil and dog tumours are independently evolved, features that circumvent the host immune system in both tumours appear necessary for evolution of cancer transmissibility. Further functional genomic research will help determine which gene(s) in these regions are most related to DFTD resistance in devils.

Taken together, our results suggest that there has been a substantial genomic response in very few generations (∼4 in Narawntapu and West Pencil Pine; ∼6 in Freycinet based on a 2-year generation time^6^,^8^), since arrival of DFTD. This finding is remarkable in light of several factors that make it difficult to detect signatures of selection on such a short timescale. The extremely recent appearance of the disease means that selection most likely acts on standing genetic variation rather than on new mutations. As a result, selection signatures in the form of allele frequency shifts may be muted and power to detect such ‘soft' selective sweeps is low. In addition, extremely low levels of genetic diversity in the Tasmanian devil make it difficult to identify a dense set of genetic markers across the genome in LD with potential causal polymorphism. Moreover, targets of selection and their degree of LD with assayed markers may be population-specific. Finally, populations have been infected at different numbers of generations in the past and thus allele frequency changes in one population may not yet be detected in another.

Given that the cancer arising from DFTD is almost universally lethal within 6 months and that prevalence rapidly reaches over 50% in reproductive age animals^8^, the selective pressure imposed by DFTD is very high. Overall, our results reflect a rapid evolutionary response to this strong selection imposed by DFTD, and such a response to a highly lethal, novel pathogen has rarely, if ever been documented in wild populations. The only other well-studied example, the evolution of rabbit resistance to myxomatosis following its release in Australia, took place over a much larger number of host generations^22^. Declines of Tasmanian devils are concerning not only due to their iconic nature, but also because losses of this apex predator have caused major shifts in trophic cascades across Tasmania^23^. Similarities of our identified host candidate genes with those that are affecting dog immunity in CTVT^20^, a much older cancer, suggest promising future research avenues for understanding the evolution of cancer transmissibility. CTVT is generally not lethal to dogs, perhaps due to co-evolutionary dynamics that have occurred in over 11,000 years since its origin^4^,^20^. Future research will focus on functional verification of the gene(s) in the two chromosomal regions experimentally *in vitro*, as well as assessments of the relationships between different allelic variants and phenotypic variables such as duration of survival with DFTD. In addition, disease-free individuals with the selectively favoured genotypes across these loci can be bred to enhance the genetic diversity of the off-island captive assurance population, in case devil reintroductions are needed in the future. Overall, the evolutionary response of Tasmanian devils observed here suggests hope for the continued survival of this species.

## Methods

### Overview of data collection

**Figure 1:**
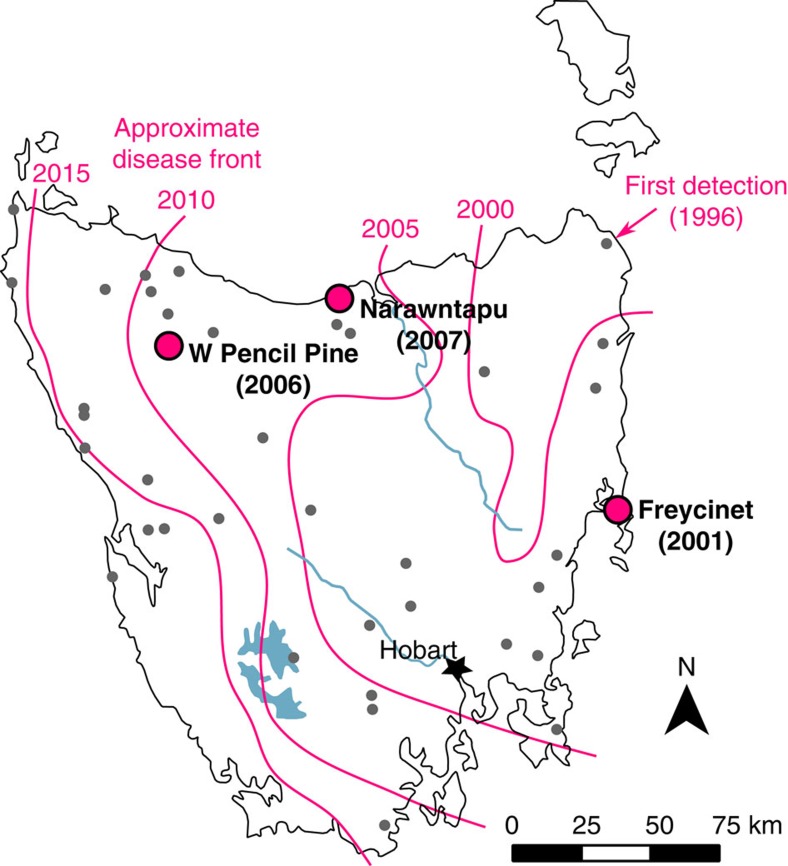
Sample collection sites in Tasmania. The three focal populations are labelled and marked with large magenta circles; smaller grey circles mark additional sampling sites, which were sampled across the entire geographic range to capture a species-wide representation of genetic diversity. The magenta lines indicate the approximate location of the disease front^6^,^7^ in 2000, 2005, 2010 and 2015.

**Figure 2:**
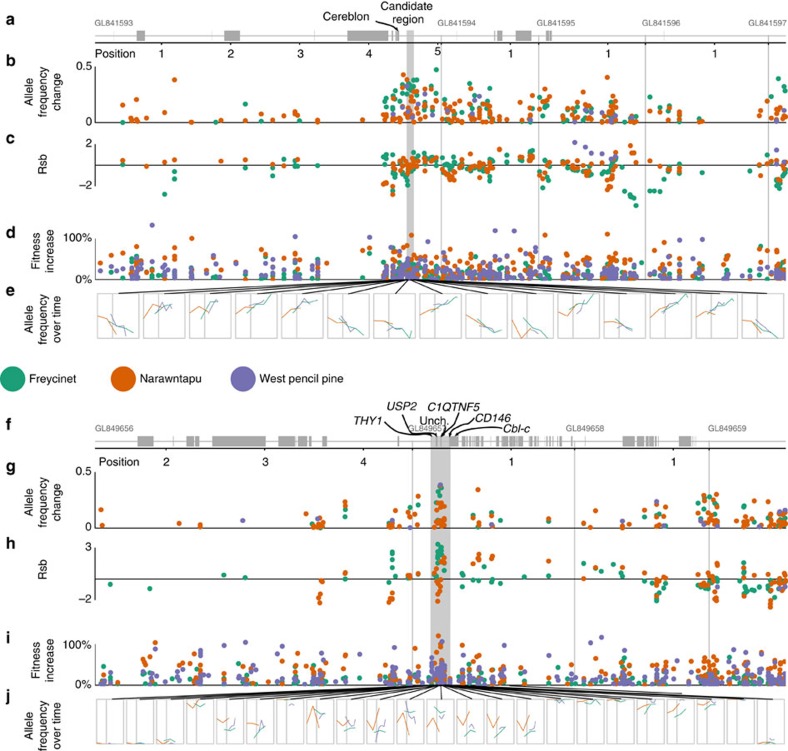
Selection test statistics for each SNP in the candidate regions and approximately 4 Mb on either side. Panels (**a**–**e**) are for chr. 2 and panels (**f**–**j**) are for chr. 3. The scaffolds, positions and genes (grey boxes) are shown in panels **a** and **f**, the positions are given in Mb from the start of each scaffold, which are marked with light grey vertical lines and a label (GL841593, and so on). Below those panels, values of three statistics are shown for each filter-passing SNP: (**b**,**g**) allele frequency change; (**c**,**h**) rsb; and (**d**,**i**) point estimates of the fitness advantage of the increasing allele. Panels **e** and **j** show the trajectory of allele frequency change over time; for clarity, we only show SNPs with relatively high genotyping rates and the *x* axis is time since detection of DFTD (first detection of DFTD is marked with a vertical line). SNPs are colour-coded by population, the candidate region is marked with a dark grey box and the names of candidate genes are labelled. Due to multiple steps of data filtering, each population has a different set of SNPs.

The methods used are depicted graphically in Supplementary Fig. 3. Tissue samples from 360 individuals were collected from 39 localities across Tasmania between 1999 and 2014 (Fig. 1; Supplementary Data 1). IRB approval was obtained for tissue collection (Washington State University Institutional Animal Care and Use Committee protocol ASAF #04392). From 36 sites, one or two samples were collected per locality. A total of 294 samples were collected from three localities—Freycinet (92 samples), Narawntapu (80 samples) and West Pencil Pine (122 samples; Supplementary Fig. 4). These samples were chosen to get a minimum of 20 samples per time point of our analyses, a standard approach for population genetics analyses. We refer to these three sites as the ‘focal populations.' The Freycinet site is a 160 km^2^ area incorporating the Freycinet Peninsula, which is on the east coast of Tasmania. DFTD was first detected in Freycinet in 2001. The Narawntapu site is Narawntapu National Park, which is in north-central Tasmania. DFTD was first detected in Narawntapu in 2007. The West Pencil Pine site is private timber land in northwestern Tasmania. DFTD was first detected at West Pencil Pine in 2006, but has impacted populations more slowly than at other sites, probably due to initial infection with a tetraploid cancer strain that was later replaced by a diploid strain in 2011 (ref. 6). Because samples could be separated into pre-DFTD arrival and post-DFTD arrival in each population, no randomization or blinding among treatments was necessary. For each sample, a single-digest RAD library was prepared with the restriction enzyme *pstI* using standard methods^11^, sequenced on an Illumina HiSeq 2500 with either paired-end or single-end 150 bp reads, and aligned to the reference genome^24^ after quality filtering and removal of PCR duplicates (Supplementary Fig. 3).

We genotyped all samples using the Stacks^25^ pipeline. After genotyping, we filtered out X chromosome SNPs, potential confounded paralogues (based on heterozygosity), SNPs with mean allele frequency (MAF) <0.01 (across all 360 samples) and SNPs that were genotyped in less than one-third of the total samples. Subsequent analyses focused on the focal populations and include some additional filtering. The following four sections provide additional details on the workflow, including specific program settings, and readers uninterested in this level of detail can skip to ‘Allele frequency changes.'

### Details of sequencing

For 72 samples (one or two from each of the 39 sampling localities), three lanes of paired-end 150 bp reads were generated from individually barcoded libraries using a HiSeq 2500. The remaining 288 samples were individually barcoded, multiplexed in pools of 96 samples, and for each pool, six lanes of single-end 150 bp reads were obtained. For two of the pools, the RAD library prep was performed with both 12-PCR cycles and 14-PCR cycles; for these two pools, three lanes of sequencing were obtained for the 12-cycle library and three lanes for the 14-cycle library. All but six of the samples from the focal populations were sequenced as part of the 288-sample single-end-read sequencing run.

From the 72-sample run, we obtained 456,639,846 read pairs; after de-multiplexing, quality filtering, PCR-duplicate removal and removal of low-quality alignments, we had 30,290–3.6 million reads per sample. For the other 288 samples, we obtained a total of 2,649,033,674 reads, or 60,000–14.7 million reads per sample (mean=6.1 million; Supplementary Data 1) after de-multiplexing, quality filtering and removal of low-quality alignments. Quality filtering removed 20% of reads, PCR duplicate removal removed 50% of the remaining reads (among just the 72 samples with paired-end data) and 20% of the reads were removed due to low mapping quality (MAPQ<40). We did not explicitly remove any individuals due to low coverage. Mean coverage of RAD loci found in at least one-third of the samples was ∼6 × across all individuals and ∼9 × when including only the individuals genotyped at each locus (Supplementary Fig. 5). (See below for details of filtering and alignment.) For all analyses, we used the Murchison *et al*.^24^ genome assembly as the reference genome^24^, and the Ensembl Devil 7.0 annotation available from Biomart^26^.

### Details of data processing and genotyping

We processed the sequencing data using the Stacks (v1.20) pipeline. For those samples with data from both 12-PCR-cycle and 14-PCR-cycle library preps, the data from different PCR cycles were processed separately until calling SNPs. We first de-multiplexed the reads and removed poor quality reads with process_radtags from the Stacks pipeline (using the -q option; we also ‘rescued' RAD-tags and barcodes with the -r option; all other settings were left at the defaults). Then for the samples with paired-end data, we removed PCR duplicates with clone_filter; this is not possible with single-end read data. Reads were aligned to the reference genome using bowtie2 (ref. 27) with the following options: --sensitive, --end-to-end, -X 900. We processed the resulting alignment files with samtools^28^. We then used custom python scripts to remove reads with a mapping quality <40, and to keep only the first read from the paired-end data for consistency among samples. We then called SNPs and produced Plink^28^ format output files using the standard Stacks reference-aligned pipeline: pstacks, cstacks, sstacks and populations. The default settings were used for Stacks except that the minimum stack depth per individual (-m) was set to 3, the bounded error model was used with an upper bound of 0.1 (--bound_high 0.1), the locus catalogue was created by matching to genomic position (-g), and we initially used any locus that was present in at least 1% of the individuals (-r 1 in the populations program). Owing to computational limits, we were not able to run the rxstacks pipeline for population-based correction of SNPs.

### Details of filtering

For all further analyses, we filtered out SNPs that were on scaffolds assigned to the X chromosome, SNPs on RAD loci where any SNP had an observed heterozygosity >0.5 across all the samples (to eliminate confounded paralogues), SNPs present in less than one-third of the samples (<120 samples) and SNPs with an MAF<0.01 across all the samples; across the entire set of 360 individuals, this resulted in 111,659 SNPs. The intention of the MAF filter was to remove SNPs that were the result of sequencing errors; thresholds set to less than 0.01 had a very marked excess of rare variants. After applying these filters, we conducted further analyses on just the samples from the focal populations, and for most analyses removed SNPs that were genotyped in less than one-third of the individuals in a particular population. With these filters, median proportions of SNP loci genotyped per individual were 69% in Freycinet, 56% in Narawntapu and 64% in West Pencil Pine. Within each population, we imposed additional filters for certain analyses.

### Details of phasing

For analyses requiring phased data, we used fastphase (v1.4.0; ref. 29). We ran fastphase on each of the focal populations separately, with 20 random starts (-T20). Each chromosome was run separately, and scaffolds were concatenated in order according to Murchison *et al*.^24^. We only included SNPs that were genotyped in at least one-third of the samples in the target population, we did not impute missing genotypes (-g option), and we chose to minimize switching error.

### Allele frequency changes

To identify SNPs and associated genes that had extreme allele frequency changes in response to DFTD, we focused on samples collected before or during the first year that cancer was detected in a population and samples from the most recent collection period. For Freycinet, we tested for allele frequency differences between 1999 and the combination of 2012 and 2013; for Narawntapu, the difference between the combination of 1999 and 2004, and 2009; and for West Pencil Pine, 2006 and the combination of 2013 and 2014. Time points were combined to increase the sample size, which allowed more accurate allele frequency estimates and increased the number of SNPs. Within each population separately, we imposed additional filters for the allele frequency change analysis: each SNP had to be genotyped in at least one-third of the samples at the beginning time point and one-third of the samples at the end time point. The exceptions to this criterion were the end time point in Freycinet and the beginning time point in West Pencil Pine, for which we required SNPs to be genotyped in at least one-half of the individuals (due to small sample sizes; Supplementary Table 2). We then pruned SNPs based on linkage disequilibrium: for every pair of SNPs within 20 SNPs and 50 kb on the same scaffold, we removed one SNP if the *R*^2^ value >0.99. *R*^2^ was calculated on unphased data by using the method implemented in Plink^30^, assuming that runs of Ns within each scaffold were accurate estimates of the gap size between contigs. This resulted in 19,639 SNPs in Freycinet, 39,378 SNPs in Narawntapu and 5,107 SNPs in West Pencil Pine.

For each population, we ranked the SNPs by allele frequency change, and identified the regions and annotated protein coding genes within 100 kb of the top 2.5% of SNPs using bedtools^31^. Those genes identified in all three populations (though not necessarily by the same SNPs in every population) were considered candidate regions based on strong LD at this genomic scale. Although it is noted that loci correlated with disease resistance could evolve independently in single populations, we restricted our candidate loci to those that evolved in all the three populations. Previous genetic studies have shown that K=2 island-wide (that is, there are two genetic clusters of devils across Tasmania^9^) and that our three focal sites are all part of the same genetic cluster, so that these three populations may share standing genetic variation for resistance that was present before DFTD. Focusing only on loci showing a signature in all the three populations minimizes issues with false-positive signatures of selection within any single population, and is thus a conservative approach. Some populations contained more than one SNP within a single candidate region; the two candidate regions contained nine unique SNPs in the top 2.5% of the distribution in at least one population (Supplementary Table 2). We repeated the analysis using the mean allele frequency change across 200 kb sliding windows (50 kb step) but did not identify any additional candidate regions across the devil genome.

### Time series selection estimates

In addition to searching for SNPs with large allele frequency changes after DFTD introduction, we also used the method of Mathieson and McVean^20^ to estimate the strength of selection on each SNP within each population by testing for changes in allele frequency over time. We applied this method to SNPs genotyped in at least one-third of the target population (Freycinet: 88,676 SNPs; Narawntapu: 83,826; West Pencil Pine: 94,003), assumed a generation time of 2 years, and assumed an effective population size of 34 for Freycinet, 37 for Narawntapu and 26 for West Pencil Pine. For Freycinet and West Pencil Pine, all time points were used, but for Narawntapu, only 2004 and 2009, which bracket the first detection of DFTD, were used. Effective population sizes were estimated using NeEstimator^32^ (v2.01) with the Jorde and Ryman two-sample temporal method^33^ to calculate *N*_e_. The 95% confidence limits for *N*_e_ obtained by jacknifing over loci were 32.3–35.8 (Freycinet), 35.5–38.9 (Narawntapu) and 23.7–28.2 (West Pencil Pine).

### Linkage disequilibrium decay

To determine the rate of LD decay in the Tasmanian devil genome, we calculated the correlation between genotypes at pairs of sites using Plink^30^. This method produces results similar to the standard *R*^2^ measure of linkage disequilibrium, but does not require phased data. Only SNPs with MAF>0.05 in the target population were used. In every population, mean linkage disequilibrium persisted substantially above the background level to at least 100 kb (Supplementary Fig. 1).

To detect potential selective sweeps that occurred during the onset of infection in the three focal populations, we calculated the Rsb statistic^12^ on phased data using the R package rehh^34^. In addition to the filters we imposed before phasing, we also excluded scaffolds with <10 SNPs, we only used SNPs genotyped in at least one-third of individuals in the target population and time point, and only haplotypes with at least a 30% genotyping rate were retained. First, for each population separately, the extended haplotype homozygosity statistic was calculated for each SNP, and integrated over genomic distance to obtain an integrated EHH (iES). Then, the natural log of the ratio of iES in the pre-infection time points to iES in the post-infection time points was calculated and standardized by subtracting the median and dividing by the standard deviation. Rsb scores greater than zero indicate that the extent of haplotype homozygosity increased after the introduction of DFTD—an indication of a selective sweep. Rsb scores were only calculated for SNPs with MAF≥0.05 both before and after infection.

### Composite test statistic

The composite test statistic, adapted from Grossman *et al*.^13^ was calculated by dividing the genome into 100 kb non-overlapping windows and selecting the SNP with the maximum quantile for Rsb and allele frequency change (separately for each population and statistic). Then, we raised the maximum quantile value to the power of the number of SNPs in the window and subtracted from one to get an adjusted *P* value for the window for each statistic:


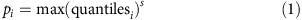


where *p*_*i*_ is the adjusted *P* value for statistic/population combination *i*, and *s* is the number of SNPs in the window with non-missing values for *i*. This is equivalent to the probability of getting a value this extreme in a sample of *s* SNPs, assuming the SNPs are independent. Because SNPs are likely not independent within each window, this adjustment is conservative. The composite score was calculated using the formula for Fisher's method for combining *P* values:


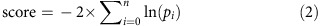


where *n* is the number of population/statistic combinations with values for the window. We then calculated a combined *P* value for each window by comparing the composite score to a chi-squared distribution with degrees of freedom equal to twice the number of statistics.

### Data availability

The sequence data has been deposited at NCBI under BioProject PRJNA306495 (http://www.ncbi.nlm.nih.gov/bioproject/?term=PRJNA306495) and BioSamples SAMN05250006-05250365. The genotype data has been deposited at Dryad under doi:10.5061/dryad.r60sv. Any other relevant data is contained within the Article and its Supplementary Files or is available from the authors upon request.

## Additional information

**How to cite this article:** Epstein, B. *et al*. Rapid evolutionary response to a transmissible cancer in Tasmanian devils. *Nat. Commun.* 7:12684 doi: 10.1038/ncomms12684 (2016).

## Supplementary Material

Supplementary Information Supplementary Figures 1-5, Supplementary Tables 1-2 and Supplementary Reference

Supplementary Data 1 Read counts, barcodes, and accession numbers for all samples

## Figures and Tables

**Table 1:** The seven candidate genes found in or closest to regions with concordant signatures of selection.

Ensembl ID	Chr	Scaffold	Human orthologue ensembl ID	Description	Putative function or phenotype
ENSSHAG00000008028	3	GL849657	ENSG00000110395	Cbl proto-oncogene, E3 ubiquitin protein ligase (*CBL-c*)	Cancer risk and immune reaction to cancer
ENSSHAG00000007967	3	GL849657	ENSG00000076706	Melanoma cell adhesion molecule (*CD146, MCAM*)	Immune regulation
ENSSHAG00000007202	3	GL849657	ENSG00000223953	C1q and tumour necrosis factor-related protein 5 (*C1QTNF5*)	Cell adhesion, retinal degeneration
ENSSHAG00000006515	3	GL849657	ENSG00000036672	Ubiquitin-specific peptidase 2 (*USP2*)	Cell-cycle regulation
ENSSHAG00000005936	3	GL849657	ENSG00000154096	Thy-1 cell surface antigen (*THY1, CD90*)	Immune regulation, cell-cycle regulation
ENSSHAG00000007088	3	GL849657	ENSG00000235718, ENSG00000259159	Uncharacterized protein	Unknown; homology to membrane-frizzled proteins
ENSSHAG00000018867	2	GL841593	ENSG00000113851	Cereblon (*MRT2, CRBN*)	Myeloma therapy target; limb and brain development
