# Supplementary material for: Rapid evolutionary response to a transmissible cancer in Tasmanian devils
Source: Nat Commun. 2016 Aug 30;7:12684. doi: 10.1038/ncomms12684 (PMC5013612; doi:10.1038/ncomms12684)
Supplement: Supplementary Information — Supplementary Figures 1-5, Supplementary Tables 1-2 and Supplementary Reference [file ncomms12684-s1.pdf]

Supplementary Information

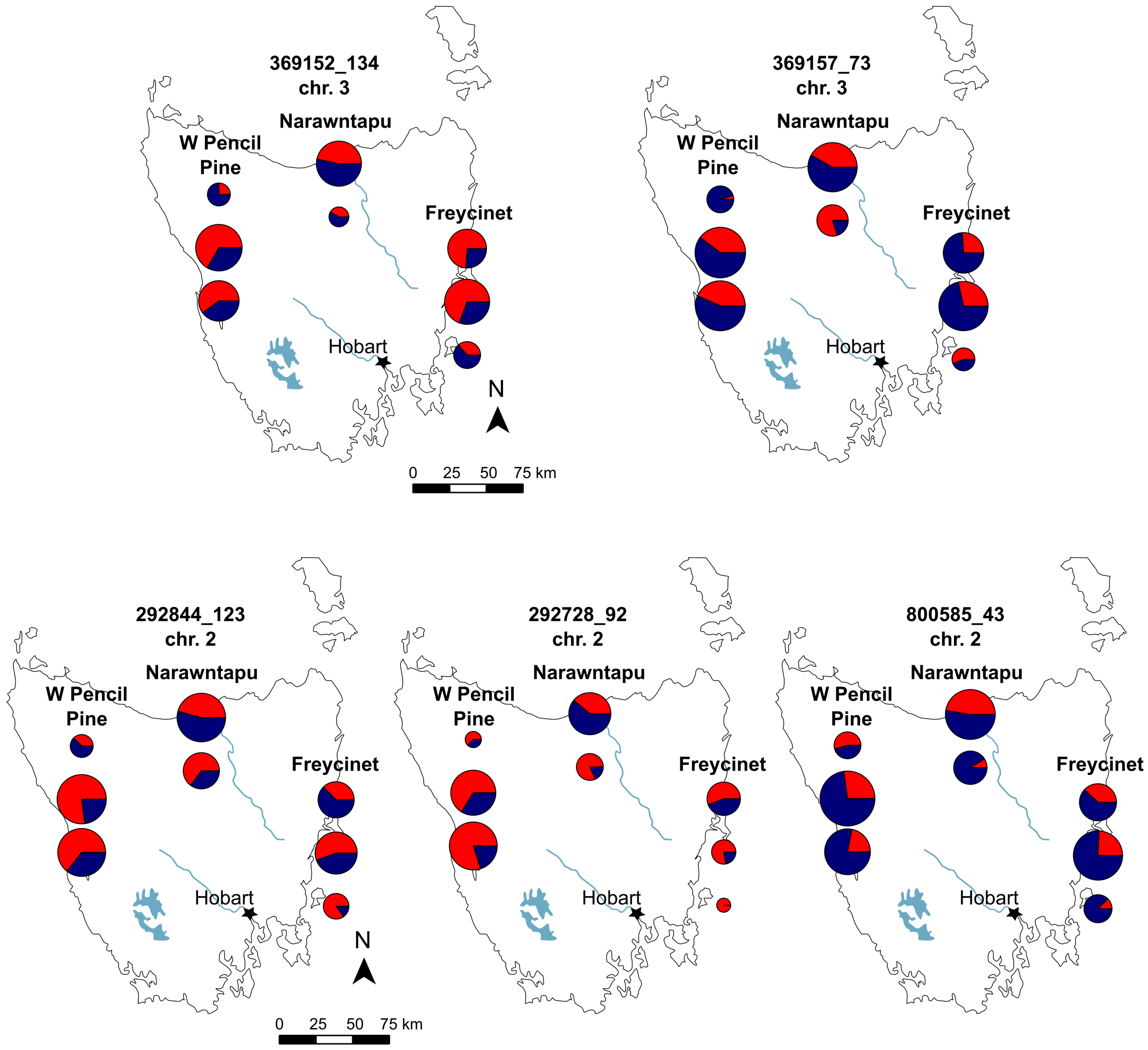

Supplementary Figure 1. Allele frequency changes of candidate SNPs by location Allele frequencies of the candidate region SNPs with the largest allele frequency changes in each of the three focal populations before, shortly after DFTD, and several years after DFTD. Time progresses from top to bottom within each population, and the (arbitrary) reference allele is colored red. The time points were 1999 (before), 2004 and 2008 (3 -7 years after DFTD), and 2012 and 2013 (11 - 13 years after) for Freycinet; 1999 and 2004 (before), and 2009 (2 years after) for Narawntapu; and 2006 (before), 2009 and 2011 (3 - 5 years after), and 2013 and 2014 (7 - 8 years after) for West Pencil Pine. Sample sizes are indicated by the area of the pie . The background map is made with Natural Earth and is in the public domain.

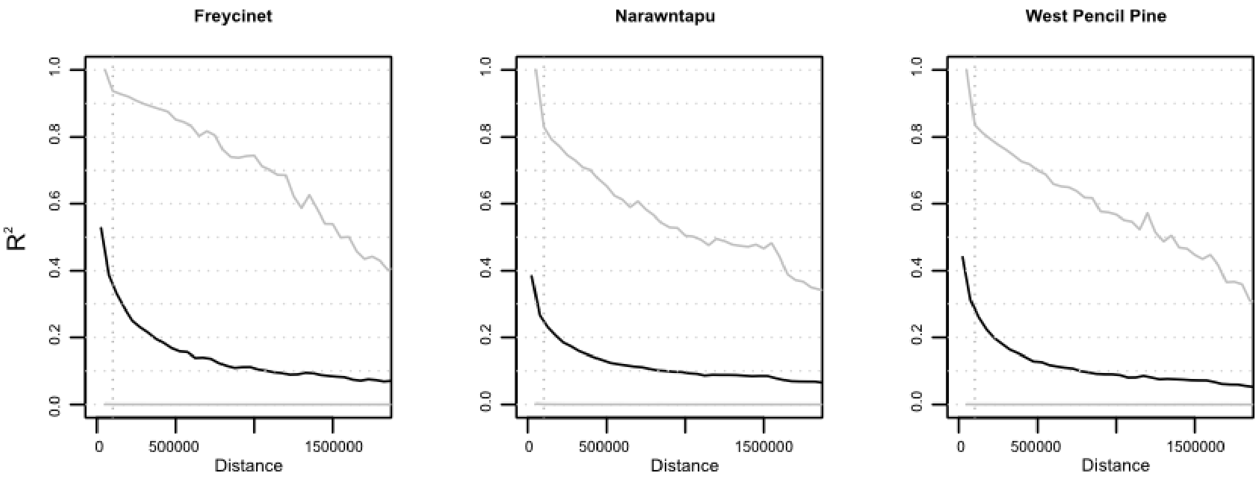

Supplementary Figure 2. LD Decay R^2 values (measures of linkage disequilibrium) calculated using Plink for 50 kb bins. The dark black line is the mean R^2 for SNPs separated by a certain distance; the grey lines are the 2.5 and 97.5 percentiles. A dotted vertical line is drawn at 100 kb.

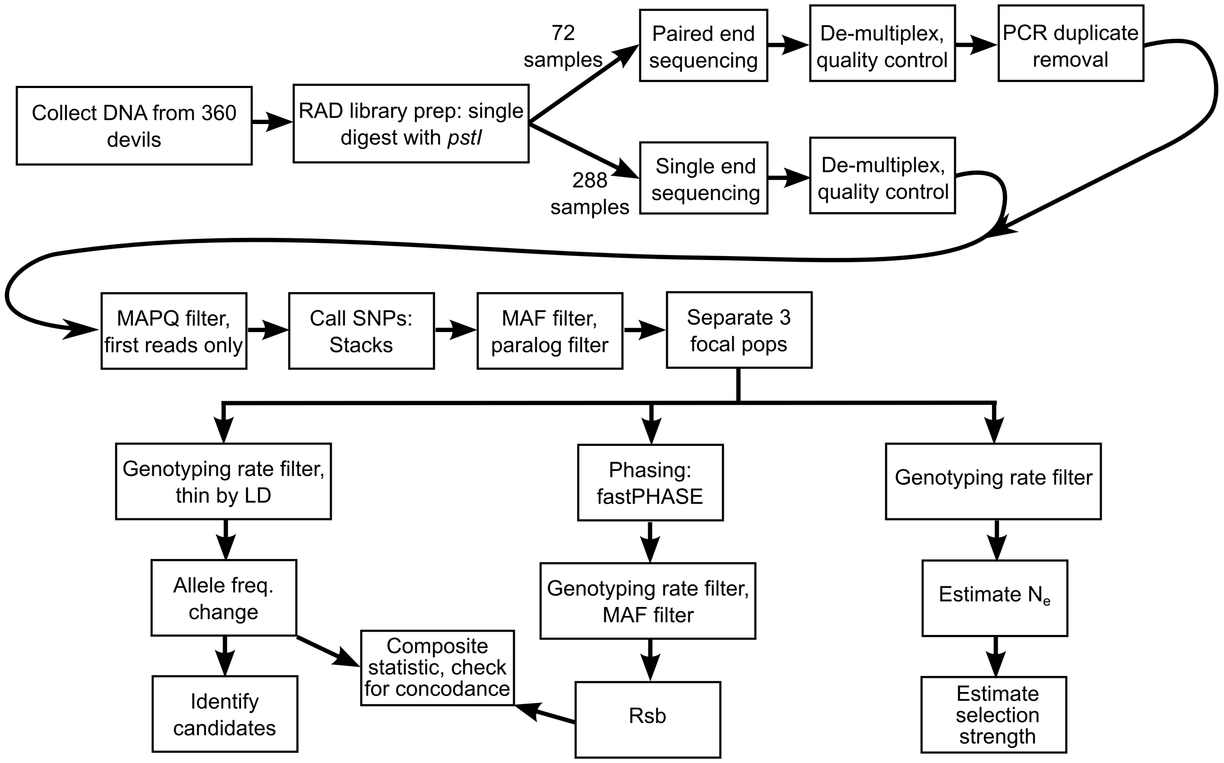

Supplementary Figure 3. Outline of the major steps in the workflow analyses See the Methods text for additional detail.

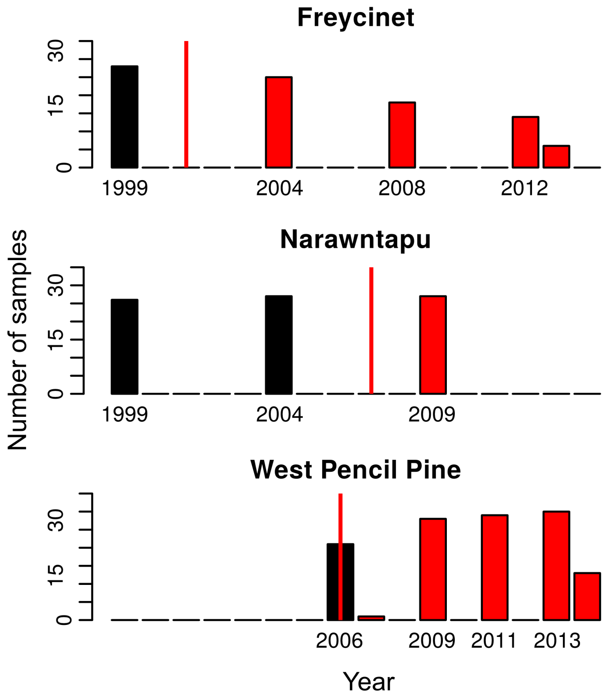

Supplementary Figure 4. Sample sizes Number of samples in each of the focal population separated by year of collection. The year in which DFTD was first detected is marked with a red line, years considered to be before DFTD in the analysis are shown in black, and years after DFTD are shown in red.

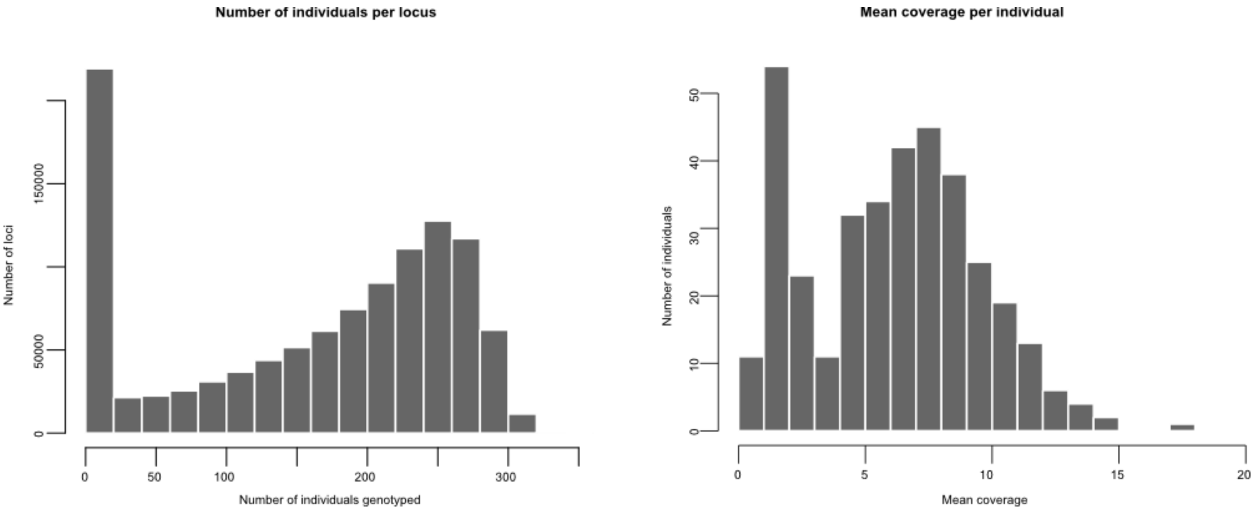

Supplementary Figure 5. Coverage distributions Left: Distribution of the number of individuals genotyped for all loci and all 360 individuals. Right: Distribution of mean coverage per individual for loci genotyped in at least one-third of all individuals.

Supplementary Table 1. Pearson's correlations coefficients and p-vaules for allele frequency changes and Rsb values between pairs of populations, and the correlation of allele frequency changes and Rsb within each .

		Allele frequency change		
SNPs				
Population 1	Population 2	r	p	df
Freycinet	Narawntapu	0.11	**< 0.001**	15,367
Freycinet	W. Pencil Pine	0.01	0.55	3,223
Narawntapu	W. Pencil Pine	0.04	**0.006**	4,176
Windows				
Freycinet	Narawntapu	0.15	**< 0.001**	8,223
Freycinet	W. Pencil Pine	0.02	0.50	806
Narawntapu	W. Pencil Pine	0.07	**0.02**	971
		Rsb		
SNPs				
Freycinet	Narawntapu	0.002	0.81	22,027
Freycinet	W. Pencil Pine	-0.02	0.60	986
Narawntapu	W. Pencil Pine	-0.17	**< 0.001**	1,029
Windows				
Freycinet	Narawntapu	0.01	0.19	11,424
Freycinet	W. Pencil Pine	-0.007	0.87	577
Narawntapu	W. Pencil Pine	-0.22	**< 0.001**	592
		Allele frequency change and Rsb		
SNPs				
Freycinet		0.02	**0.03**	12,862
Narawntapu		-0.004	0.50	23,207
W Pencil Pine		0.001	0.98	984
Windows				
Freycinet		-0.03	**0.02**	7,809
Narawntapu		-0.09	**< 0.001**	12,432
W Pencil Pine		0.03	0.51	375

Supplementary Table 2. SNPs in the two candidate genomic regions that had allele frequency changes at or greater than the 97.5 percentile, and the population in which the large allele frequency changes were detected. For each SNP, we give the absolute magnitude of allele frequency change, as well as the quantile within the population and the start and end frequencies, and we give the point estimate and 95% CI for the fitness advantage conferred by the increasing allele[1].

SNP Id	Scaffold: position	Population	Allele frequency change (start – end), quantile	Fiteness advantage value (95% confidence interval)
369152_134	chr3_GL849657: 278112	Freycinet	0.38 (0.29 – 0.58), 0.98	0.25 (-0.07 – 0.57)
369163_5	chr3_GL849657: 292515	Freycinet	0.36 (0.14 – 0.5), 0.98	0.27 (-0.07 – 0.60)
292763_85	chr2_GL841593: 4649090	Freycinet	0.38 (0.42 – 0.04), 0.98	0.32 (-0.02 – 0.67)
292774_62	chr2_GL841593: 4695526	Freycinet	0.38 (0.46 – 0.08), 0.98	0.32 (-0.04 – 0.70)
292844_123	chr2_GL841593: 4979736	Freycinet	0.48 (0.63 – 0.15), 0.99	0.29 (-0.03 – 0.60)
369157_73	chr3_GL849657: 283671	Narawntapu	0.38 (0.42 – 0.80), 0.995	1.01 (0.33 – 1.70)
800580_43	chr2_GL841593: 4488810	Narawntapu	0.31 (0.58 – 0.27), 0.99	0.33 (-0.34 – 0.93)
292728_92	chr2_GL841593: 4501785	Narawntapu	0.43 (0.61 – 0.18), 0.998	0.79 (0.11 – 1.43)
800585_43	chr2_GL841593: 4553946	Narawntapu	0.39 (0.47 – 0.09), 0.996	0.83 (-0.17 – 1.56)
369157_43	chr3_GL849657: 283701	West Pencil Pine	0.39 (0.05 – 0.43), 0.99	0.43 (-0.06 – 0.94)
369157_73	chr3_GL849657: 283671	West Pencil Pine	0.39 (0.05 – 0.43), 0.99	0.44 (-0.06 – 0.96)
800585_43	chr2_GL841593: 4553946	West Pencil Pine	0.33 (0.55 – 0.22), 0.98	0.44 (-0.07 – 0.93)

Supplementary References

1. Mathieson, I. & McVean, G. Estimating selection coefficients in spatially structured populations from time series data of allele frequencies. *Genetics* **193,** 973–984 (2013).
